# Complicated cutaneous leishmaniasis caused by an imported case of *Leishmania tropica* in Japan: a case report

**DOI:** 10.1186/s41182-021-00312-4

**Published:** 2021-03-06

**Authors:** Hiroyuki Kitano, Chizu Sanjoba, Yasuyuki Goto, Kazumasa Iwamoto, Hiroki Kitagawa, Toshihito Nomura, Keitaro Omori, Norifumi Shigemoto, Michihiro Hide, Yoshitsugu Matsumoto, Hiroki Ohge

**Affiliations:** 1 Department of Infectious Diseases, Hiroshima University Hospital, 1-2-3 Minamiku Kasumi, Minamiku, Hiroshima City, Hiroshima 734-8551 Japan; 2 Laboratory of Molecular Immunology, Department of Animal Resource Sciences, Graduate School of Agricultural and Life Sciences, Tokyo University, 1-1-1 Yayoi, Bunkyo-Ku, Tokyo, Tokyo 113-8657 Japan; 3 Department of Dermatology, Graduate School of Biomedical & Health Sciences, Hiroshima University, 1-2-3 Minamiku Kasumi, Minamiku, Hiroshima City, Hiroshima, 734-8551 Japan

**Keywords:** Cutaneous leishmaniasis, Japan, Skin diseases, Pakistan, Imported diseases, *Leishmania tropica*

## Abstract

**Background:**

Leishmaniasis is not endemic in Japan, and imported cases are rare. However, there are increasing concerns regarding imported cases of cutaneous leishmaniasis from endemic countries to Japan. This report describes a case of imported cutaneous leishmaniasis that was diagnosed and treated in Japan.

**Case presentation:**

A 53-year-old Pakistani man presented with skin lesions on both malleoli of his right ankle and the dorsum of the left foot. The skin lesions manifested as erythematous nodules surrounding an ulcer in the center of the lesion. The lesions of the malleoli of his right ankle each measured 3 × 3 cm, and the lesion on the top of his left foot measured 5 × 4 cm. He had been living and working in Japan but had a history of a visit to Pakistan for about 2 months in 2018. The skin lesions were biopsied. Giemsa and hematoxylin and eosin staining of biopsy samples showed amastigotes of *Leishmania* in macrophages, and the presence of *Leishmania* was confirmed by skin tissue culture. Polymerase chain reaction using biopsy specimens identified *Leishmania* parasites, and DNA sequence analysis revealed that the species was *Leishmania tropica*. The patient was treated with intravenous liposomal amphotericin B for 6 days. The erythema disappeared, and the erythematous nodules resolved within 3 weeks.

**Conclusion:**

This is the first report of imported cutaneous leishmaniasis caused by *L. tropica* from Pakistan, and it is interesting that all three testing modalities showed positive results in this case.

## Background

Leishmaniasis is a major global health problem. An estimated 0.7–1 million new cases of leishmaniasis are reported per year from approximately 100 endemic countries [1]. Leishmaniasis is a vector-borne parasitic disease caused by protozoa of the genus *Leishmania* and is transmitted between mammalian hosts by female sandflies [2, 3]. Leishmaniasis is classified into three main clinical syndromes: cutaneous leishmaniasis (CL), mucocutaneous leishmaniasis, and visceral leishmaniasis [4]. The most predominant form of leishmaniasis is CL, with an estimated global incidence of 600,000 to one million cases each year [5]. CL is not life-threatening but has a profound socioeconomic impact due to the stigmatization of infected and cured individuals, as the disease may leave residual disfiguring scars [6, 7]. Leishmaniasis is not endemic in Japan, and there have been few reports of imported infections; approximately 27 imported cases were reported from 1950 to 1995 [8–10], and only 3 imported CL cases were reported in a few articles since 1996 [11–13]. However, there are increasing concerns of imported cases of CL from endemic countries to Japan [11]. We report a case of CL caused by *Leishmania tropica* in an adult male who presented to the Hiroshima University Hospital in Hiroshima, Japan. The infection was confirmed by immunohistochemistry, skin tissue culture, and polymerase chain reaction (PCR).

## Case presentation

**Fig. 1:**
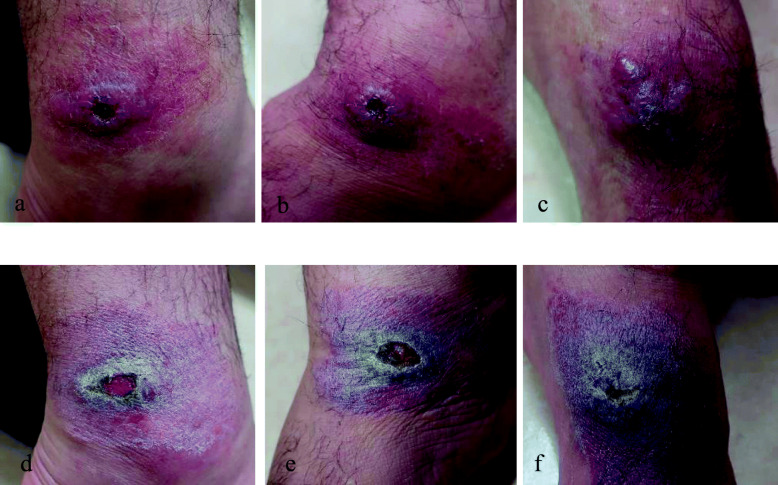
Skin lesions at initial presentation (**a**–**c**) and after treatment (**d**–**f**). Erythematous nodules with peripheral ridges and central ulceration located on the right medial malleolus (**a**), right lateral malleolus (**b**), and the top of the left foot (**c**). After treatment, the erythema has disappeared and the nodules on the right medial malleolus (**d**), right lateral malleolus (**e**), and top of the left foot (**f**) have resolved

**Fig. 2:**
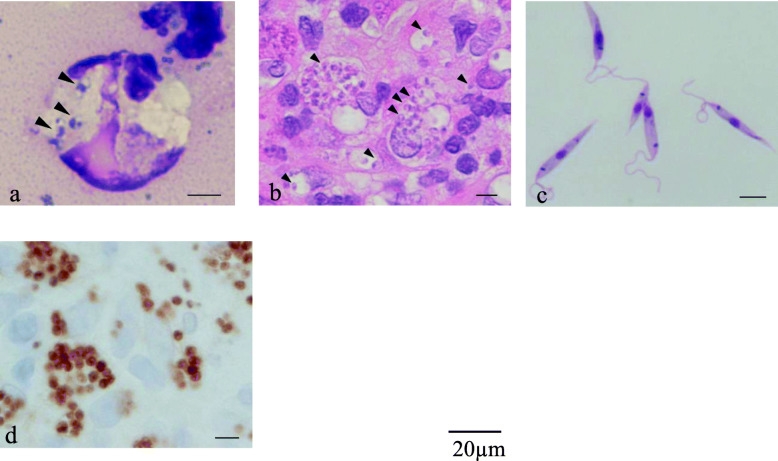
Appearance of the lesions on histology. Giemsa staining (**a**) and hematoxylin and eosin staining (**b**) of punch biopsy specimens show *Leishmania* amastigotes (arrowheads). Giemsa staining of the skin tissue culture shows *Leishmania* promastigotes (**c**). C11C antibody-positive cells in the patient’s skin lesion (**d**). All bars are 5 μm

**Fig. 3:**
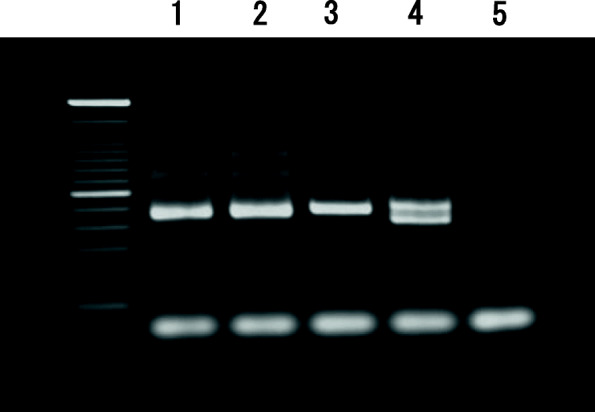
Polymerase chain reaction results. Amplified DNA extracted from a skin sample is positive (lane 1). The positive control samples with *Leishmania tropica* DNA, *L. major* DNA, and *L. donovani* DNA are shown in lanes 2, 3, and 4, respectively, and the negative control is shown in lane 5

**Fig. 4:**
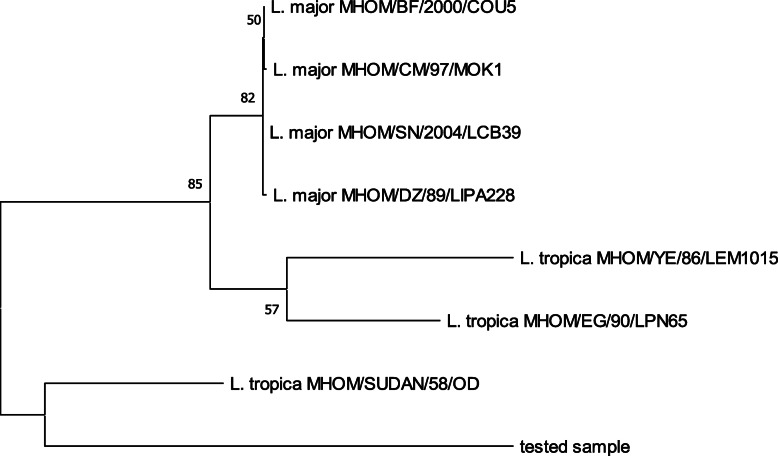
The phylogenetic tree. The phylogenetic tree was constructed based on the 368-nt nucleotide sequences by the maximum likelihood method and Kimura 2-parameter model

A 53-year-old man from Pakistan presented to the Hiroshima University Hospital in Japan in January 2019 with persistent skin lesions of both malleoli of his right ankle and the dorsum of his left foot. The immunohistochemistry of a skin biopsy, performed in November 2018 at another hospital in Japan, had suggested leishmaniasis. The lesions and itching had appeared in November 2018 and did not respond to gentamicin ointment. Three years previously, he had been living and working in Japan but returned to Khyber Pakhtunkhwa province, Pakistan, which is an endemic area for CL caused by *L. tropica* and *Leishmania major* [14–16] for about 2 months (August–September) in 2018. The skin lesions manifested as erythematous nodules surrounding an ulcer in the center of the lesion. The lesions on the malleoli of his right ankle each measured 3 × 3 cm, and the lesion on the dorsum of his left foot measured 5 × 4 cm (Fig. 1a–c). He requested further tests for an accurate diagnosis of his skin disease. We performed a punch biopsy of each skin lesion and collected the exudate from the skin lesions. Giemsa staining of the exudate and hematoxylin and eosin (HE) staining of the skin biopsy tissue demonstrated *Leishmania* amastigotes in macrophages on histological examination (Fig. 2a–b). *Leishmania* promastigotes were detected 2 weeks later in the culture of a skin punch biopsy specimen that had been preserved in saline and then transferred to medium 199 supplemented with 10% heat-inactivated fetal bovine serum (Fig. 2c). Immunohistochemical staining using the C11C antibody (a monoclonal antibody against *Leishmania* peroxiredoxin/thiol-specific antigen) [17] also detected positive cells in the skin lesion (Fig. 2d). For the molecular diagnosis of *Leishmania* infection, DNA was extracted from the skin biopsy sample using a DNeasy Blood and Tissue Kit (QIAGEN, Tokyo, Japan). We performed PCR with the DNA samples to identify the *Leishmania* parasite mini-exon gene [18] and detected products of expected sizes on agarose gel electrophoresis. The causative species was identified as either *L. major* or *L. tropica* (Fig. 3). The amplified DNA fragments were size selected from agarose gels and cloned into the vector pCR2.1-TOPP, continuously subjected to sequencing. The phylogenetic tree was constructed based on the 368-nt nucleotide sequences by the maximum likelihood method and Kimura two-parameter model using Molecular Evolutionary Genetics Analysis Version 10.2.2 (MEGA X) software [19]. The bootstrap scores were calculated for 1000 replicates (Fig. 4). The patient consented to all the specialized diagnostic tests, including DNA sequence analysis that was performed for species identification. Based on these findings, we diagnosed the lesions as CL caused by *L. tropica* and started treatment with intravenous liposomal amphotericin B (AmBisome, Dainippon Sumitomo Pharma, Osaka, Japan), which was administered intravenously daily for 6 days. The patient did not experience fever or fatigue or any other side effects during the course of treatment. Three weeks after starting treatment, the erythema disappeared, and the erythematous nodules resolved (Fig. 1d–f).

## Discussion and conclusion

Leishmaniasis is endemic in many developing countries. One study found that CL represented 3.3% of skin lesions in 4594 returning travelers with travel-related skin disorders, worldwide [20]. Most of the returning travelers had traveled to Latin America, and 15% had become infected within 2 weeks of staying in an endemic country [20]. In Japan, there is currently no evidence of the presence of the species of sandfly that transmit leishmaniasis. Considering the ecology of sandflies, it is unlikely that sandflies will enter Japan with travelers and expand their habitat. Although the possibility of the disease spreading to Japan is low, notification of imported cases is necessary in the age of globalization.

The major causative parasite species of CL are *L. major* and *L. tropica* endemic to the Mediterranean Basin, the Middle East, the Horn of Africa, and the Indian subcontinent [21]. In Pakistan, CL is caused by either *L. major* or *L. tropica* [22], as in this case. Leishmaniasis may be underdiagnosed or overdiagnosed, and unnecessary treatment has been administered in some cases in Pakistan [23].

Nodular lesions of CL are often mistaken for furuncles [1]; therefore, the diagnosis should be made based on careful examination and the presence of a typical lesion in conjunction with an appropriate history of exposure [23].

Parasitological confirmation should be sought preferably by confirming the growth of the organism in culture [12]. A full-thickness biopsy from an infiltrated margin of the lesion is useful for histological examination and culture [23]. Specimens collected by biopsy are stained using HE and Giemsa stains and examined for *Leishmania* amastigotes in the skin tissue [24]. However, it is not always possible to make the diagnosis based on a skin biopsy in clinical practice. The parasite may not be detected even by the most adequate methods [23]. PCR testing is more sensitive than immunohistochemistry and culture and is useful for confirming the diagnosis [23]. In our case, PCR targeting a mini-exon gene could not distinguish between *L. major* and *L. tropica* as the causative species, and hence, we performed DNA sequencing and determined that the causative organism was *L. tropica*. Serology may not be helpful for the diagnosis of CL because antibodies tend to be undetectable or present in low titers [23]; however, serological testing can be useful for initial screening [25].

The treatment of CL involves either intralesional injection of 8.5% meglumine antimonite (Glucantime) or intravenous liposomal amphotericin B [26]. Liposomal amphotericin B was approved for the treatment of leishmaniasis in June 2009 in Japan. As the patient had three skin lesions and was experiencing severe symptoms, we chose to use intravenous liposomal amphotericin B (3 mg/kg, 250 mg/body, administered daily for 6 days) as recommended by the Japanese guideline **(**https://www.nettai.org).

In this case, *Leishmania* amastigotes were detected in HE- and Giemsa-stained skin biopsy specimens, and *Leishmania* promastigotes were detected by skin culture. In cases of CL, it is rare for all three testing modalities to be positive. This is the first report of imported CL caused by *L. tropica* from Pakistan and it contributes useful information on the most appropriate anti-leishmanial therapies for imported cases of CL.

## Data Availability

All data generated or analyzed during this study are included in this published article.

## Abbreviations

CL: Cutaneous leishmaniasis
HE: Hematoxylin and eosin
*L. major*: *Leishmania major*
*L. tropica*: *Leishmania tropica*
PCR: Polymerase chain reaction
